# When ultrarapid is ultrarapid: on importance of temporal precision in neuroscience of language

**DOI:** 10.3389/fnhum.2015.00576

**Published:** 2015-10-21

**Authors:** Yury Y. Shtyrov, Tatyana A. Stroganova

**Affiliations:** ^1^Center of Functionally Integrative Neuroscience (CFIN), Institute for Clinical Medicine, Aarhus UniversityAarhus, Denmark; ^2^Centre for Cognition and Decision Making, NRU Higher School of EconomicsMoscow, Russia; ^3^Moscow MEG Center, Moscow State University for Psychology and EducationMoscow, Russia

**Keywords:** neocortex, semantics, magnetoencephalography, time course, brain, language comprehension

**This opinion responds to the commentary by Papeo and Caramazza (2014)**.

The mechanisms through which our brain generates, stores, and invokes complex semantic representations, such as those used in language, remain unknown. One key question is whether the basic brain structures controlling movements and perceptions directly participate in higher-order cognitive processes. Their involvement in semantic representations of individual words is therefore hotly debated in current literature. Clarifying the mechanisms of this involvement was the goal of our previous research (Shtyrov et al., 2014), critically analyzed by the above commentary. Using magnetoencephalography, we found ultra-rapid (commencing ~80 ms after the disambiguation point) activations and deactivations in the motor cortex (defined non-invasively using MRI and localizer task in MEG) in response to unattended action-related verbs and nouns, with words related to different body-parts (*kick, swallow, throw*) selectively activating corresponding somatotopic representations, while suppressing word-incompatible motor representations. In our view, these instant activation patterns, which emerged for different words types in the absence of focused attention on the stimuli, advocate automatic involvement of sensorimotor circuits in word comprehension.

Among other things, the above commentary raises a range of important questions:

- It rightly identifies the timing as a crucial feature of the effects discovered. However, it criticizes the timing of the effects as being locked to the disambiguation points, rather than word onsets.
- The earliness of semantic effects is questioned for being simultaneous with acoustic-sensory processes.
- The critique assumes that the disambiguation point only disambiguates grammatical class, but not semantic content. After re-calculating the effects to the word onset, the commentary concludes that they are post-comprehension.
- A question is raised with respect to the motor system involvement in referentially underspecified use of action language.

Below, we highlight important considerations related to these neurolinguistic experimental issues:

- The timing of the effects as stemming from the word disambiguation/recognition points is indeed a crucial feature that should, in our view, be implemented in any neurolinguistic experiment. Often in such experiments, large stimulus sets are compared, with average responses being used to make conclusions on all words of a certain category. First, this creates physical stimulus variance, when different stimulus types have diverging physical features (duration, frequency, etc.); this is especially difficult to control in the auditory modality when spoken stimuli unfold over time with different dynamics. Differences even in basic physical features may lead to differential brain activation (Näätänen and Picton, 1987) that could overlap with, mask, cancel, or be misinterpreted as language-related effects. Second, this creates psycholinguistic variance, when stimuli diverge in their linguistic features, including word recognition parameters in spoken words. The latter may be especially difficult to control, as different words become uniquely recognized at different times, in extreme cases shortly after their onset or only after a substantial post-offset period (Marslen-Wilson, 1987). The conventional approach of matching average parameters across stimulus categories can help mitigate these problems, but still has a caveat: for any small and short-lived effects (as all known early ERP peaks are, as well as any transient effects outside local maxima), the variance in the stimulus group may reduce or even remove any effects in the average responses, particularly if time-locked to the word onset. While later deflections (e.g., N400, P600) are smeared by such averaging but, being large in amplitude and long-lasting, still survive it, this strategy could be fatal for capturing the earliest short-lived transient small-scale activity. Therefore, to capture the entire neural dynamics of language processing, it is important to (1) maximally reduce stimulus variance, e.g., by using a fixed set of tightly controlled stimuli, and (2) time-lock electrophysiological response to key psycholinguistic markers in the auditory stream, most importantly—to the point in time, when the available information allows for differentiating the stimulus from other similar sounds and, ultimately, for identifying it.
  The commentary specifies the time of “~200 ms after the word onset” as an upper limit for considering motor system's involvement in comprehension as direct. While 200 ms is indeed often considered the borderline between initial automatic and late top-down controlled stages of language processing, we argue that, *for spoken words*, it is not “after the word onset” that this timing should be calculated from. In fact, 200 ms is approximately the duration of one short CV-syllable usually devoid of any meaning. Instead, more linguistically relevant time markers should be used, such as the word recognition point when the information available is sufficient for confident identification, or at least the disambiguation point, when the perceptual input noticeably diverges between a few competitors.
  A few studies illustrate the efficiency of this approach. For example, in a study using large groups of words and pseudowords in an N400 design, no reliable lexical effects were found when time-locking ERPs to the word onsets (Friedrich et al., 2006). However, when ERPs were realigned to disambiguation points, marked N400 effects were found—moreover, they commenced already before 200 ms. In another recent study comparing groups of words and pseudowords, the tight control over recognition points led to the discovery of transient neuromagnetic lexicality effects around 50–80 ms that cannot be easily identified otherwise (MacGregor et al., 2012).
- The commentary questions the earliness of semantic effects based on the assumption that these latencies are associated with sensory acoustic analysis. True, acoustic variables can *still influence* brain responses at these latencies (e.g., loudness effects on N100 are well known), but the extraction of acoustic features commences much earlier. Acoustic information transfer from the cochlea to temporal neocortex only takes ~10–20 ms (Eldredge and Miller, 1971; Rupp et al., 2002) with basic acoustic feature extraction taking place at 20–50 ms (Krumbholz et al., 2003; Lutkenhoner et al., 2003). Even the earliest marked cortical deflections around ~50 ms (in the P50/P1 range) have been linked to higher-level cognitive information processing (Palva et al., 2002; Yadon et al., 2009; MacGregor et al., 2012, 2014). Crucially, P50 generators (at least as defined by non-invasive neuroimaging tools) are distributed beyond primary auditory areas, including parietal, cingulate, and frontal associative cortices (Boutros et al., 2013) refuting the possibility that it merely reflects an acoustic feature extraction stage. Interestingly, to support the notion of sensory-only processing at sub-100 ms latencies the commentary cites a seminal review by Friederici (2002); the very same research group have, however, published a series of studies claiming high-level syntactic processes already at 40–60 ms (e.g., Herrmann et al., 2009, 2011a,b). Combined, this evidence suggests that one should not be surprised by semantic effects as late as 80–120 ms. In fact, the available conduction time estimates and electrophysiological findings suggest that the earliest linguistically-relevant cortical processes might commence around 30–60 ms.
- The commentary assumes that the disambiguation point only disambiguated the stimulus's grammatical category (verb/noun) but not semantics, and that the semantic information is available much earlier, already during the stimulus onset. This is a misunderstanding of crucial experimental design features. The vast majority of stimuli were meaningless pseudowords fully sharing their onsets with the critical verbs and nouns. Thus, if the semantic information were available during the onset, the similar semantically-specific motor activations should also take place for the frequent pseudowords as, up to the disambiguation point, they were identical to the words. This clearly did not happen: motor-cortex effects were only present for the real words *after* they became distinct from the meaningless fillers. The reported effects were found in the *difference response* between activation to rarely presented (“deviant”) words and frequent (“standard”) pseudowords, i.e., the mismatch negativity component triggered by a contrast between them^1^. There was no sufficient semantic information in the onsets to identify the meaning of either word before the disambiguation point: not only could these onsets end as meaningless pseudowords, but the onsets themselves have either no meaning if presented standalone or carry a meaning unrelated to the full form (therefore, the analogy “brΛs” = “throw-” in the commentary is incorrect). In principle (although less relevant in a repetitive oddball design), these onsets also have numerous other completions unrelated semantically to the stimulus words. Taken together, these factors rule out any certain identification of semantics before the disambiguation point.
  As mentioned, the commentary relies on a sequential model of language comprehension, which considers latencies before 100 ms sensory-related (Friederici, 2002). The very same model, however, also suggested that semantic information is only processed at 300–500 ms, in line with M350 and N400 research (Embick et al., 2001; Stockall et al., 2004; Kutas and Federmeier, 2011). Thus, even if the logic of recalculating brain responses to the word onset were correct and the effects were indeed in the classical N400 time range, the very same classical framework would place them together with the rest of lexico-semantic dynamics, and not with post-comprehension phenomena.
  Crucially, other studies have also indicated early motor cortex involvement in word comprehension. We will not repeat reviews of such findings (e.g., Pulvermüller and Fadiga, 2010), but would instead like to highlight the importance of investigations using visual word presentation. Auditory modality (the “native” modality of the language function) presents experimenters with serious challenges as the stimulus unfolds in time and the amount of available acoustic and linguistic information changes continuously and rapidly. In contrast to this, in the visual domain, sensory information about the word is available instantaneously in its entirety. Using visually-presented words could therefore help disentangle the earliest stages of neural word access, by avoiding the complications of time-locking brain responses to dynamically changing input. Indeed, visual word reading investigations using EEG and MEG suggested early (within 200 ms *after the word onset*) activation of the motor system in semantic access (Hauk and Pulvermüller, 2004; Hauk et al., 2008; Boulenger et al., 2012), even for action words of participants' second language (Vukovic and Shtyrov, 2014). Although satisfying the 200-ms threshold, these latencies are substantially later than the sub-100 ms dynamics under discussion. This could be explained by various factors. First, information transfer from the visual system to the temporo-frontal core language network may lead to inevitable delays in language-circuitry activations. Second, previous visual investigations focused largely on ERP/ERF peaks, whereas we scrutinized time periods outside local maxima—and our effects were, indeed, found before the absolute response peak. Third, in the auditory modality one cannot exclude a degree of predictive processing when word-initial information allows partial pre-activation of corresponding memory traces before word completion, as suggested by the Cohort model of speech comprehension (Marslen-Wilson and Tyler, 1980); this latter possibility also partially aligns with the commentators' critique of our findings.
- Papeo and Caramazza raise an important question of underspecified motor semantics (“throw a party”). Whereas the original study was *not* set up to address modality-specific brain systems' involvement in the comprehension of metaphoric or idiomatic language, the “embodied cognition” framework does not refute the existence of representations without a direct motor (or another modality-specific) reference as such; one example could be function words. On the other hand, words are naturally acquired in the context of experiencing the objects, actions and concepts they represent. This, in the Hebbian associative learning framework, leads to establishing distributed cortical representations which may therefore include modality-specific structures. Once established, nothing prevents the use of these circuits for a variety of purposes whenever the word they represent is called upon. This may include non-literal use of action words such as in “throw a party,” “kick the bucket,” or “swallow one's pride,” with obscure or even absent action connotation. There is, however, a dearth of studies on this topic. At least one MEG experiment suggested early (150–200 ms) activation of the motor system in idiom comprehension (Boulenger et al., 2012). Further investigations are essential to answer the question of modality-specific contributions to language comprehension in non-literal contexts.
  Finally, the commentary appears to have missed a crucial element of our experimental design: the attention-distraction paradigm. The participants were asked to concentrate on non-linguistic visual input and ignore the sounds; no linguistic task or any word-related activities were required. While this design does not fully prevent a degree of active word processing, this removal of stimulus-related task and even attention on stimuli does minimize the risk on covert imagery or simulation necessary for late post-comprehension processes to take place. A number of studies that manipulated attention on linguistic stimuli indicated that the earliest stages of language processing are automatic and largely resilient to top-down control (Hahne and Friederici, 1999; Pulvermüller et al., 2008; Garagnani et al., 2009; Shtyrov, 2010; Kimppa et al., 2015). Further investigations are needed in order to validate this automaticity explicitly in the lexical semantics domain, for example, by manipulating task demands and attention levels.
  On a more general note, we should also point out that most of data currently available on the subject (including the study under discussion) are based, with exception of a handful of patient studies, on non-invasive measures of brain activity whose neuroanatomical precision remains limited. Thus, further research is necessary to validate tentative motor cortical generators active in semantic processing using more precise tools, such as direct electocorticography recordings (ECoG). Such experiments (e.g., Mesgarani et al., 2014; Steinschneider et al., 2014) are becoming instrumental in detailing rapid cortical timecourse of language comprehension (including the motor cortex involvement in speech perception, Chang et al., 2011); their extension to studies of cortical dynamics related to (motor) semantics is a fruitful future direction.
  In conclusion, we would like to stress the importance of experimental investigations into the language comprehension timecourse and of fruitful theoretical debates of the kind sparkled by the commentators, to whom we are grateful for a critical and focused discussion of our findings. Fast neuroimaging modalities are indispensable in comprehensive investigations of this timecourse. For these investigations to be meaningful, the issue of time-locking must be taken into account most rigorously. Precisely defining and orthogonally modulating acoustic onsets and offsets, physical make-up, disambiguation and word recognition points, as well as validating any effects using different modalities of stimulation (auditory, visual) and data acquisition (MEG, EEG, ECoG, TMS, f/sMRI, and their combinations) are, in our view, a prerequisite for the success of future neurolinguistic experiments.

## Conflict of interest statement

The authors declare that the research was conducted in the absence of any commercial or financial relationships that could be construed as a potential conflict of interest.

## Footnotes

^1^The mismatch negativity paradigm is a sensitive tool for registering word-specific memory-trace activations while strictly controlling for acoustic factors by employing identical standard-deviant acoustic contrasts in different contexts; (Pulvermüller and Shtyrov, 2006; Näätänen et al., 2007).
